# Supplementary material for: A one-year hospital-based prospective COVID-19 open-cohort in the Eastern Mediterranean region: The Khorshid COVID Cohort (KCC) study
Source: PLoS One. 2020 Nov 5;15(11):e0241537. doi: 10.1371/journal.pone.0241537 (PMC7644058; doi:10.1371/journal.pone.0241537)
Supplement: S1 File — (PDF) [file pone.0241537.s001.pdf]

S1 File. The specification of the KCC based on STROBE Statement.

Objectives:

1- To study COVID-19 disease resulting from hospitalization

2- To determine risk factors associated with COVID-19 morbidity and mortality

3- To determine the effect of different treatments on disease prognosis

4- To study on factors associated with readmission between COVID-19 patients

5- To study on the natural trend of disease during follow-ups

6- To study on the secondary psychological outcomes of COVID-19 outbreak

Study design: An open, prospective cohort study.

Setting: A hot zone based on COVID-19 outbreak from Iran, pointed hospital for COVID-19 patients, from February 2020 with one year follow up (it is an open cohort until 1500 recruitments).

Participants: The COVID-19 infected patients who were admitted to Khorshid hospital. Patients are followed at the time of admission by laboratory and clinical investigation, 1 week, and 4 weeks by phone and 12 weeks, and 1 year after discharge by interview based on filling valid questionnaires and spirometer checking in the hospital.

Variables

Diagnosis: Positive PCR test or typical CT with specified symptoms.

Predictors: Demographic variables, Socioeconomic status Symptoms and signs, Laboratory findings, CT imaging results, treatment (time of first order, duration, Dose), COVID-19 exposure-related variables, Smoking and Tobacco, duration of symptoms before hospital admission, and the number of infected patients in the family.

Confounders: Comorbidities, long-time medication.

Outcomes: ICU admission, Death, Ventilation, Discharge, Readmission (with the event, and time to event data), Depression, Anxiety, Stress, changes in quality of life (event number).

Data sources/ measurement: A Total of 12 training interns extracted data. Patient data were collected through the hospital's electronic medical record system and included general personal information, previous medical history, laboratory testing, blood coagulation function, blood biochemistry, and CT findings.

Biases: Selection, information, and recall bias.

Study size: We started with 600 cases, and it is an open-cohort study

Quantitative variables: The quantitative variables are the laboratory findings measured using calibrated instruments.

Mission data managements: In this paper, 490 patients with complete information were analyzed. Missing analysis in the subgroups and sensitivity analysis are performed when the entire samples are used.
