# Supplementary material for: A one-year hospital-based prospective COVID-19 open-cohort in the Eastern Mediterranean region: The Khorshid COVID Cohort (KCC) study
Source: PLoS One. 2020 Nov 5;15(11):e0241537. doi: 10.1371/journal.pone.0241537 (PMC7644058; doi:10.1371/journal.pone.0241537)
Supplement: S1 Table — (DOCX) [file pone.0241537.s002.docx]

**S1 Table**. **Checklist of following patients admitted to the hospital.**

| Name: Family: |
| --- |
| **Sex**: Male Female |
| Date of Birth: |
| The number of family members: |
| **Occupation**: Employed Unemployed |
| Height: Weight: |
| **Education**: Illiterate College Diploma Graduated |
| **Smoking status**: Yes No |
| **Tobacco usage**: Yes No |
| Residence Address: Postal Code: |
| **Quarantine status before admission**: Yes No |
| **First Symptoms** |
| Sneeze Runny Nose Dry Cough Phlegm Cough Headache |
| Body Pain Abdominal pain Vomiting Diarrhea |
| Chest Pain Fatigue Shortness of Breath Fever |
| Duration Of having Symptoms (Days): |
| **Same symptoms In Family**: Yes No |
| **Travel History**: Where (country/City): When (Date): |
| **Getting influenza vaccine since September:** Yes No |
| Risk Factors |
| **Patients with Immunodeficiency** |
| Taking corticosteroids or other immunosuppressive drugs Malignancies  Organ transplant HIV Chemotherapy History |
| **Patients with Underlying Disease** |
| CVD Hypertension Respiratory Disease Diabetes |
| COPD CKD Dialysis history |
| Vital Symptoms: BP T PR RR O_2_Sat |
| **Laboratory Findings**:  Drug Hx: CRP: Hb: WBC: LYM: PMN:  PLT: CXR: CT scan Results: |
| Treatment Strategy |
| Outpatients Treatment: |
| Inpatients Treatment: |
| Suggested Treatment: |
