# Supplementary material for: A one-year hospital-based prospective COVID-19 open-cohort in the Eastern Mediterranean region: The Khorshid COVID Cohort (KCC) study
Source: PLoS One. 2020 Nov 5;15(11):e0241537. doi: 10.1371/journal.pone.0241537 (PMC7644058; doi:10.1371/journal.pone.0241537)
Supplement: S2 Table — (DOCX) [file pone.0241537.s003.docx]

**S2 Table**. **Checklist of following patients discharged from the hospital.**

| Name: Family: |
| --- |
| Date of discharge |
| Time of follow up First Second Third Fourth |
| Sex: Male Female |
| First Symptoms |
| Runny Nose Dry Cough Phlegm Cough Headache |
| Body Pain Abdominal pain Vomiting Diarrhea |
| Chest Pain Fatigue Shortness of Breath Fever |
| Sleep disorder Yes NO |
| Decreased appetite Yes NO |
| Reduced sense of smell (hyposmia) Yes NO |
| Weight Loss Yes NO |
